# IL-1β mediates lung neutrophilia and IL-33 expression in a mouse model of viral-induced asthma exacerbation

**DOI:** 10.1186/s12931-018-0725-z

**Published:** 2018-01-24

**Authors:** Irma Mahmutovic Persson, Mandy Menzel, Sangeetha Ramu, Samuel Cerps, Hamid Akbarshahi, Lena Uller

**Affiliations:** Department Experimental Medical Science Unit of Respiratory Immunopharmacology, BMC D12, Lund University, 221 84 Lund, Sweden

**Keywords:** Asthma exacerbation, HDM, IL-1β, IL-33, Mouse model, Neutrophilia, TSLP

## Abstract

**Background:**

Viral-induced asthma exacerbations, which exhibit both Th1-type neutrophilia and Th2-type inflammation, associate with secretion of Interleukin (IL)-1β. IL-1β induces neutrophilic inflammation. It may also increase Th2-type cytokine expression. We hypothesised that IL-1β is causally involved in both Th1 and Th2 features of asthma exacerbations. This hypothesis is tested in our mouse model of viral stimulus-induced asthma exacerbation.

**Method:**

Wild-type (WT) and IL-1β deficient (IL-1β^−/−^) mice received house dust mite (HDM) or saline intranasally during three weeks followed by intranasal dsRNA (PolyI:C molecule known for its rhinovirus infection mimic) for three consecutive days to provoke exacerbation. Bronchoalveolar lavage fluid was analysed for inflammatory cells and total protein. Lung tissues were stained for neutrophilic inflammation and IL-33. Tissue homogenates were analysed for mRNA expression of Muc5ac, CXCL1/KC, TNF-α, CCL5, IL-25, TSLP, IL-33, IL-1β, CCL11 and CCL2 using RT-qPCR.

**Results:**

Expression of IL-1β, neutrophil chemoattractants, CXCL1 and CCL5, the Th2-upstream cytokine IL-33, and Muc5ac were induced at exacerbation in WT mice and were significantly inhibited in IL-1β^−/−^ mice at exacerbation. Effects of HDM alone were not reduced in IL-1β-deficient mice.

**Conclusion:**

Without being involved in the baseline HDM-induced allergic asthma, IL-1β signalling was required to induce neutrophil chemotactic factors, IL-33, and Muc5ac expression at viral stimulus-induced exacerbation. We suggest that IL-1β has a role both in neutrophilic and Th2 inflammation at viral-induced asthma exacerbations.

**Electronic supplementary material:**

The online version of this article (10.1186/s12931-018-0725-z) contains supplementary material, which is available to authorized users.

## Background

Asthma exacerbation is episodic worsening of the asthma symptoms in patients with already on-going chronic inflammation. The frequency of exacerbations is associated with disease severity and may be a factor in aggravation of the basic respiratory disease [1]. Rhinovirus has been suggested a major cause of asthma exacerbations [2] which comprise a major patient burden. The link between asthma exacerbations and respiratory viral infection has been known for many years [3], but causative molecular mechanisms of asthma exacerbations are still not fully understood. Potential mechanisms have been suggested including exaggerated cytokine expression producing Th2- and Th1-type airway inflammation [4, 5].

Using mouse models of ovalbumin (OVA)- and house dust mite (HDM)-provoked asthma, with added challenges involving rhinovirus or its infection intermediate, dsRNA, we have previously produced experimental asthma exacerbations involving human asthma-like mixed granulocyte inflammation [6, 7]. Further, our in vivo exacerbation model involving dsRNA, similar to viral induced exacerbation in asthmatic patients [5], was associated with significant cell necrosis determined as increased bronchoalveolar lavage (BAL) fluid levels of lactate dehydrogenase (LDH) [6, 7]. We also observed that major Th2-upstream cytokines such as thymic stromal lymphopoietin (TSLP) and IL-33 were induced at exacerbation [6]. Interestingly, we demonstrated significant up-regulation of IL-1β in our asthma exacerbation model [6].

IL-1β has recently been discussed in severe asthma [8] and in relation to exacerbations of chronic obstructive pulmonary disease (COPD) and asthma [9]. The possible role of IL-1β in rhinovirus-induced exacerbations is supported by a chain of mechanisms involving pattern recognition receptors (PRRs). The infection activates PRRs to increase expression of pro-IL-1β that will be transformed into mature and active form of IL-1β by the inflammasome named nod-like receptor family pyrin domain containing-3 (NLRP3) [10, 11]. NLRP3 is reportedly increased in neutrophilic asthma [10, 12]. IL-1β is a well-recognised inducer of neutrophilia potentially contributing to the pronounced neutrophilic lung inflammation that occurs both in the human disease [13] and in animal exacerbation models [6, 7, 12]. A demonstration in human nasal fibroblasts suggested that IL-1β may increase TSLP and IL-33 gene expression and protein [14]. Little is known about the involvement of IL-1β in Th2-upstream cytokine expression at exacerbation of asthma.

We hypothesised that features of asthma exacerbation such as neutrophilic inflammation and Th2-upstream cytokines are dependent on IL-1β signalling. To test this hypothesis we have employed our HDM-based model of viral stimulus-induced exacerbation in both wild-type (WT) mice and in mice lacking IL-1β [6]. We demonstrate that inflammatory features of exacerbation involving expression of common neutrophilic cytokines as well as a major Th2-upstream cytokine depend on IL-1β signalling.

## Methods

### Animals and ethical approval

Experiments were approved by the Malmö/Lund Animal Experimental Ethics Committee at the Lund District Court in Sweden (permit no. M36–13). Eight to ten weeks old male C57BL/6 and IL-1β deficient (IL-1β^−/−^) mice with the same background were used in the study, breed and housed in Lund [15]. (The IL-1β gene expression was analysed to confirm the knockdown of IL-1β, Additional file 1: Figure S1.) Mice had free access to food and water during the experimental period.

### Experimental study design

**Fig. 1:**
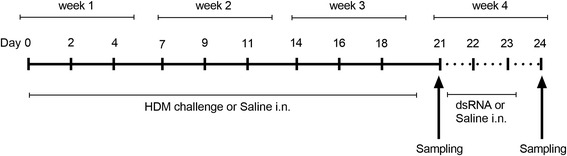
HDM-induced allergic asthma and dsRNA-triggered exacerbation in WT and IL-1β^−/−^ mice. HDM was used as a challenging agent for three weeks to induce experimental asthma in mice. Control mice received saline challenges for three weeks and both groups were terminated 72 h after the last challenge. In addition, four groups of mice continued the study and received saline control or dsRNA intranasally for three consecutive days to provoke exacerbation, in both WT and IL-1β^−/−^ mice. The dsRNA stimulus is a synthetic molecule also known as Poly(I:C) and served as a mimic of rhinoviral replication. The experiment was terminated at day 24, 24 h after the last dsRNA stimulation

To study asthma exacerbation we first performed allergen challenges with HDM inducing allergic airway inflammation using an experimental protocol previously published [6]. Briefly, the mice were challenged intranasally (i.n.) every other day during 3 weeks, with 25μg of HDM *Dermatophagoides pteronyssinus* (GREER, Lenoir, USA) or saline as control (Fig. 1). Subsequently, dsRNA challenges (100ug) using Poly(I:C) (polyinosine–polycytidylic acid) (InvivoGen, San Diego, CA, USA), mimicking a rhinoviral infection or saline (control) was administered i.n. for three consecutive days, to induce asthma exacerbation The experiment was terminated after the exacerbation phase (day 24). To see if lack of IL-1β altered the baseline allergic exudative inflammation that is present promptly after HDM exposure, some experiments were terminated after HDM exposure alone (Fig. 1). BAL was performed followed by dissection of the lungs as previously described [6, 7]. Different lung lobes were snap frozen in liquid nitrogen or perfused with- and stored in the fixative 4% formaldehyde.

### Bronchoalveolar lavage (BAL)

By performing a tracheostomy and connecting a tube to the trachea, lungs were rinsed by Saline and the solution was then collected in a tube and kept cold before processed. BAL fluid (BALF) was then spun down and supernatant was separated from cells by centrifugation, then stored in − 80 °C until use. The cell pellet was further processed, re-suspended in PBS and total cell count was measured using an automatic cell counter as previously described [7]. Cells were cytospin-centrifuged onto microscopic slides, stained with May-Grünwald and Giemsa and differentially counted. A total of 400 cells per slide were counted blindly and presented as percentage ratios containing macrophages/monocytes, lymphocytes, neutrophils or eosinophils. The BALF supernatant was used to analyse total protein concentration with bicinchoninic acid (BCA) assay kit (Pierce® BCA Protein Assay Kit detection, Fischer Scientific AB, Sweden), and also ELISA analysis was performed to determine the protein concentration of the cytokine CXCL1/KC (R&D Systems, UK) according to the manufacturer’s instructions, using standard curve based concentration determination. The detection limit for the total protein BCA assay was in the range of 5–2000 μg/mL and the CXCL1/KC ELISA kit had a detection limit in the range of 15.60–1000 pg/mL.

### Lung histology and homogenates

Paraffin embedded lung tissue was sectioned into 4 μm thick sections and stained with Haematoxylin & Eosin (H&E) to investigate general lung inflammation. Additionally, immunohistochemistry was performed. Lung tissue sections were firstly blocked using 5% serum, following overnight incubation at 4 °C with primary goat anti-mouse IL-33 antibody (R&D Systems, UK) or antibodies for detecting neutrophils (NIMP-R14; Abcam, Cambridge, UK). The sections were then rinsed and incubated with secondary anti-goat IgG antibody (R&D Systems, UK) for the IL-33 staining while neutrophil stained sections were incubated using biotinylated secondary antibodies (ABC Vectastain; Vector Laboratories, Burlingame, CA, USA). Both IL-33 and neutrophil stained sections were visualised with 3,3-diaminobenzidine (DAB, Vectastain, Vector Laboratories, USA) and followed by a Haematoxylin counterstain. Tissue sections were scanned with ScanScope (Aperio Technologies, USA) using software Aperio ScanScopeTM and representative photos were presented. Tissue staining for both neutrophils and IL-33 was quantified using the software ImageScope where positive staining (signal) was assessed by algorithms within the program set to detect brown staining and express the signal as weak positive, positive or strong positive. Only the strong positive signal was quantified and presented as “positivity of signal/mm^2^ tissue area” as relative to control sample group (WT mice, HDM/Saline group).

### Quantification of gene expression using RT-qPCR

The frozen lung lobes were homogenised using OmniPrep Rotor (Omni International, USA) with lysis buffer supplied within the RNA extraction kit RNA II (Nucleospin® RNA II kit, Machery-Nagel, Germany). RNA was extracted and 2 μg of total RNA was reverse transcribed using kit from PrimerDesign (PrimerDesign, UK). Subsequently, PCR products were detected after performed thermocycling on a sequence detection system (Stratagene, M × 3000P, La Jolla, CA, USA), and genes of interest were calculated in relation to the reference gene 18S (PrimerDesign, UK) using the ΔΔCt method [16] as previously performed [7]. The primer sequences are shown in table ‘Additional file 2: Table S1’, as supplementary data, for the following genes; Muc5ac, CXCL1, CCL5, IL-25, IL-1β and CCL11 (from Qiagen Sciences Inc., USA) and TNF-α, TSLP, IL-33 and CCL2 (from PrimerDesign, UK).

### Statistical analysis

Data are expressed as mean values and SEM, and all data were analysed using non-parametric tests using the software GraphPadPrism (version 7.03 GraphPad Software, San Diego California USA, www.graphpad.com). One-way ANOVA with multiple comparison test was used when comparing more than two groups. The two-tailed Mann–Whitney test was used to compare variance between groups. *P*-values of less than 0.05 were considered statistically significant. Comparison between WT vs. KO groups with the same stimuli were indicated with the symbol * when significant difference occurred. Comparing stimuli to control group was indicated with the symbol # when significant difference occurred.

## Results

### IL-1β^−/−^ mice exhibited reduced lung inflammation compared to WT mice at dsRNA-induced exacerbation

**Fig. 2:**
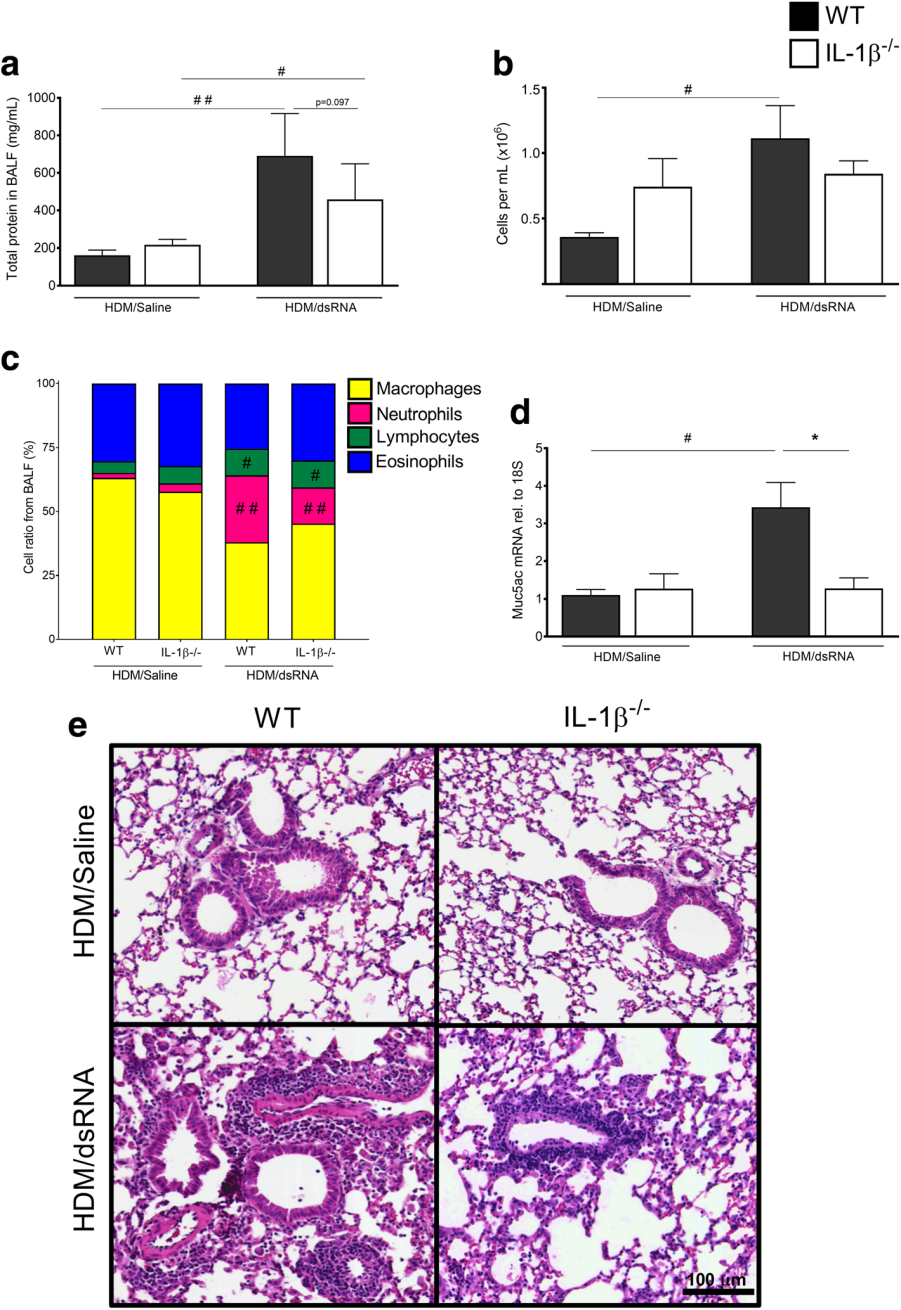
BALF and lung tissue inflammation were reduced in IL-1β^−/−^ mice at exacerbation. Total protein (**a**) and total cells (**b**) were analysed from BALF. Differential cell count in BALF was quantified from cytospinned and May-Grünwald/Giemsa-stained cells (**c**). mRNA expression of Muc5ac was quantified in lung tissue homogenates using RT-qPCR (**d**). The relative gene expression was related to the reference gene 18S and normalised to the fold change of HDM/Saline control. Data are presented as mean ± SEM, *n* = 4–13 mice in each group. ## = *p* < 0.01 compared to respective HDM/Saline control and # = *p* < 0.05 compared to respective HDM/Saline control. The comparison between WT and IL-1β^−/−^ at exacerbation is indicated by * = *p* < 0.05. Representative H&E stained lung tissue sections from both WT and IL-1β^−/−^ (**e**). Scale bar indicates 100 μm for all four micrograph images

Total protein concentration in BALF, likely reflecting plasma exudation [17], was increased at exacerbation in WT mice (*p* < 0.01) compared to WT control (HDM/Saline) group while the IL-1β^−/−^ mice showed a trend towards lower levels of total proteins at exacerbations compared to WT mice (Fig. 2a). HDM challenge alone increased immune cells and total protein in both WT and IL-1β^−/−^ mice compared to respective saline controls (Additional file 3: Figure. S2a-b). However, HDM challenge alone showed no difference in BALF total protein between WT and IL-1β^−/−^ mice as determined just after HDM exposure (Additional file 3: Figure. S2B) or after three additional days of saline exposure (Fig. 2a). Indeed, there was no tendency at all, that lack of IL-1β reduced protein and cell exudation evoked by HDM at peak responses (24 h post HDM) or at early resolution of HDM-induced inflammation (which equals to 3 days of saline treatment post HDM). Effects on BALF cells showed similar pattern to BALF total protein during exacerbation with increase in total BALF cells in the WT group (*p* < 0.05) but not in the IL-1β^−/−^ mice (Fig. 2b). There was increased percentage of lymphocytes and neutrophils in the BALF at exacerbation compared to HDM-Saline group for both WT and IL-1β^−/−^ mice (Fig. 2c). Beside immune cell activity and plasma exudation, hyper-secretion of mucus is a general feature of acute asthma [13]. Also, cell culture studies have suggested a role of IL-1β in Muc5ac expression [18, 19]. Therefore, we analysed Muc5ac mRNA expression, which was significantly increased (*p*<0.05) at exacerbation in the WT mice compared to control. However, Muc5ac mRNA expression was not induced at exacerbation in the IL-1β^−/−^ mice being significantly lower (*p* < 0.05) compared to WT mice (Fig. 2d). Histological analysis showed increased lung inflammation at exacerbation in WT mice but less pronounced in IL-1β^−/−^ mice (Fig. 2e).

### Tissue neutrophilia and neutrophil chemotactic cytokines were reduced in IL-1β^−/−^ mice at exacerbation

**Fig. 3:**
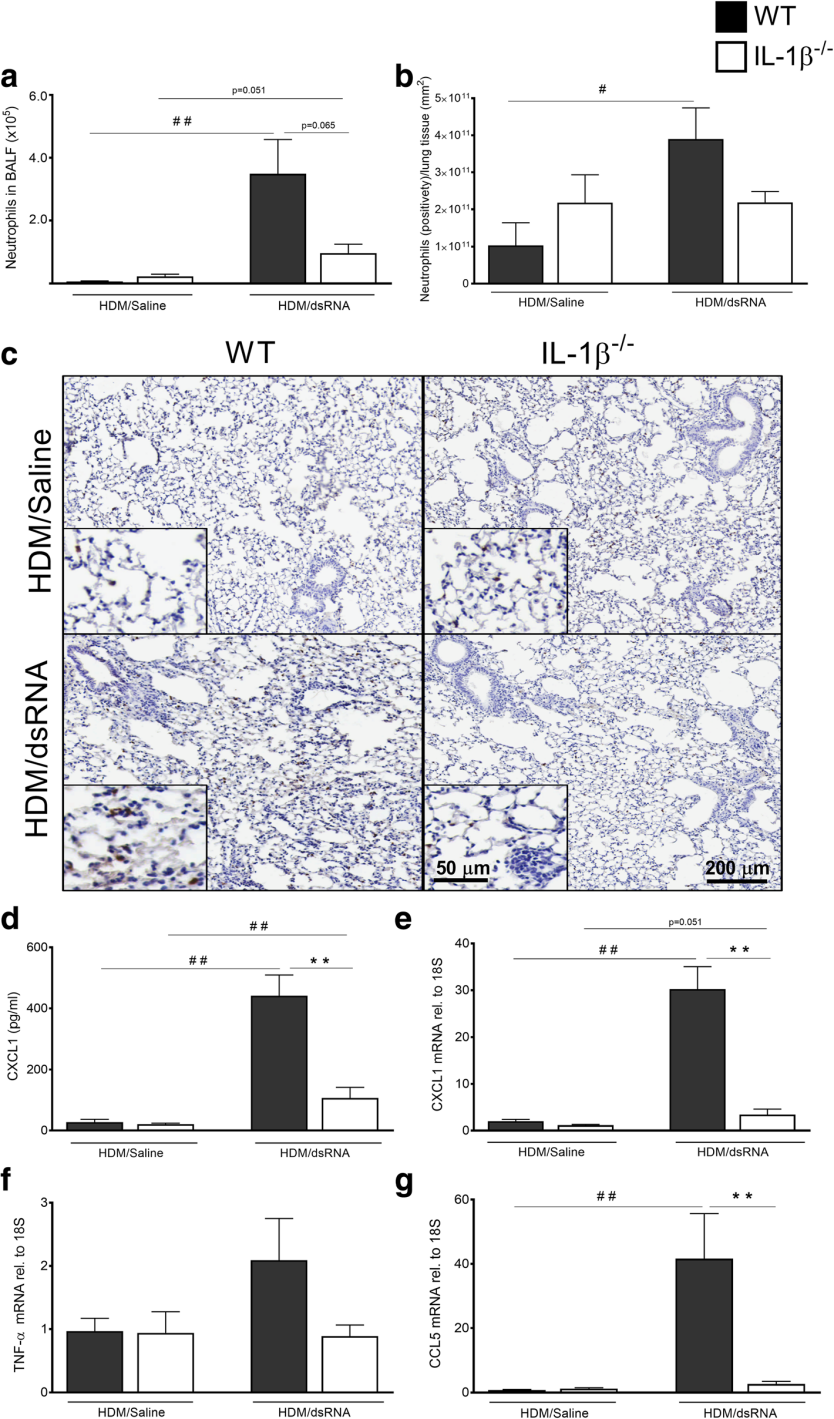
Induced neutrophilia in BALF and lung tissue was associated with induced chemokine expression at exacerbation in WT mice while reduced in the IL-1β^−/−^ mice. Airway neutrophils quantified in BALF (**a**) and lung tissue (**b**). Representative slides of immunohistochemically stained neutrophils in the lung tissue demonstrating reduced neutrophilia in IL-1β^−/−^ mice during exacerbation compared with WT mice (**c**). Scale bar in small micrographs indicate 50 μm while large micrographs scale bar is 200 μm. Levels of different neutrophil chemoattractants analysed in the lung: CXCL1 protein in BALF (**d**) and lung tissue mRNA (**e**), also lung tissue mRNA expression of TNF-α (**f**) and CCL5 (**g**). The relative gene expression was related to the reference gene 18S and normalised to the fold change of HDM/Saline control. Data are presented as mean ± SEM, *n* = 4–7 mice in each group. ## = *p* < 0.01 compared to respective HDM/Saline control and # = *p* < 0.05 compared to respective HDM/Saline control. The comparison between WT and IL-1β^−/−^ at exacerbation is indicated by ** = *p* < 0.01 or * = *p* < 0.05

dsRNA administration on top of the HDM challenges (HDM/dsRNA) induced a significant increase in BALF- and lung tissue neutrophils compared to the WT control; HDM/Saline group (Fig. 3a-c). Although both WT and IL-1β^−/−^ mice developed pulmonary neutrophilia at exacerbation, a trend towards lower BALF- and lung tissue neutrophil counts occurred in the IL-1β^−/−^ mice compared to WT mice (Fig. 3a-c). In accord, expression of neutrophil attractants was much more pronounced in WT than in IL-1β^−/−^ mice (Fig. 3d-g). The main neutrophil chemotactic protein, CXCL1/KC was induced at exacerbation in WT mice to a greater extent than in IL-1β^−/−^ mice, both at protein level (*p* < 0.01) and gene level (*p*<0.01), (Fig. 3d-e). TNF-α was not induced in WT and IL-1β^−/−^ mice whereas RANTES/CCL5, was increased at exacerbation to a greater extent in WT mice than in IL-1β^−/−^ mice (Fig. 3f-g). In addition, the other immune cells analysed from BALF, and tissue gene expression of chemoattractants CCL2 (macrophage chemoattractant) and CCL11 (eosinophil chemoattractant) did not seem to differ between the WT and the IL-1β^−/−^ mice at exacerbation (Additional file 4: Figure S3a-e). Compared to the saline controls allergen challenge alone only induced slight increase in neutrophil counts and this effect did not differ between WT and IL-1β^−/−^ (Additional file 3: Figure 2c). Lack of IL-1β was not associated with any reduction in neutrophil counts nor neutrophilic chemoattractants in animals receiving saline post HDM exposure (Fig. 3a-b).

### IL-33 was increased at exacerbation in the lung tissue of WT mice but not in IL-1β^−/−^ mice

**Fig. 4:**
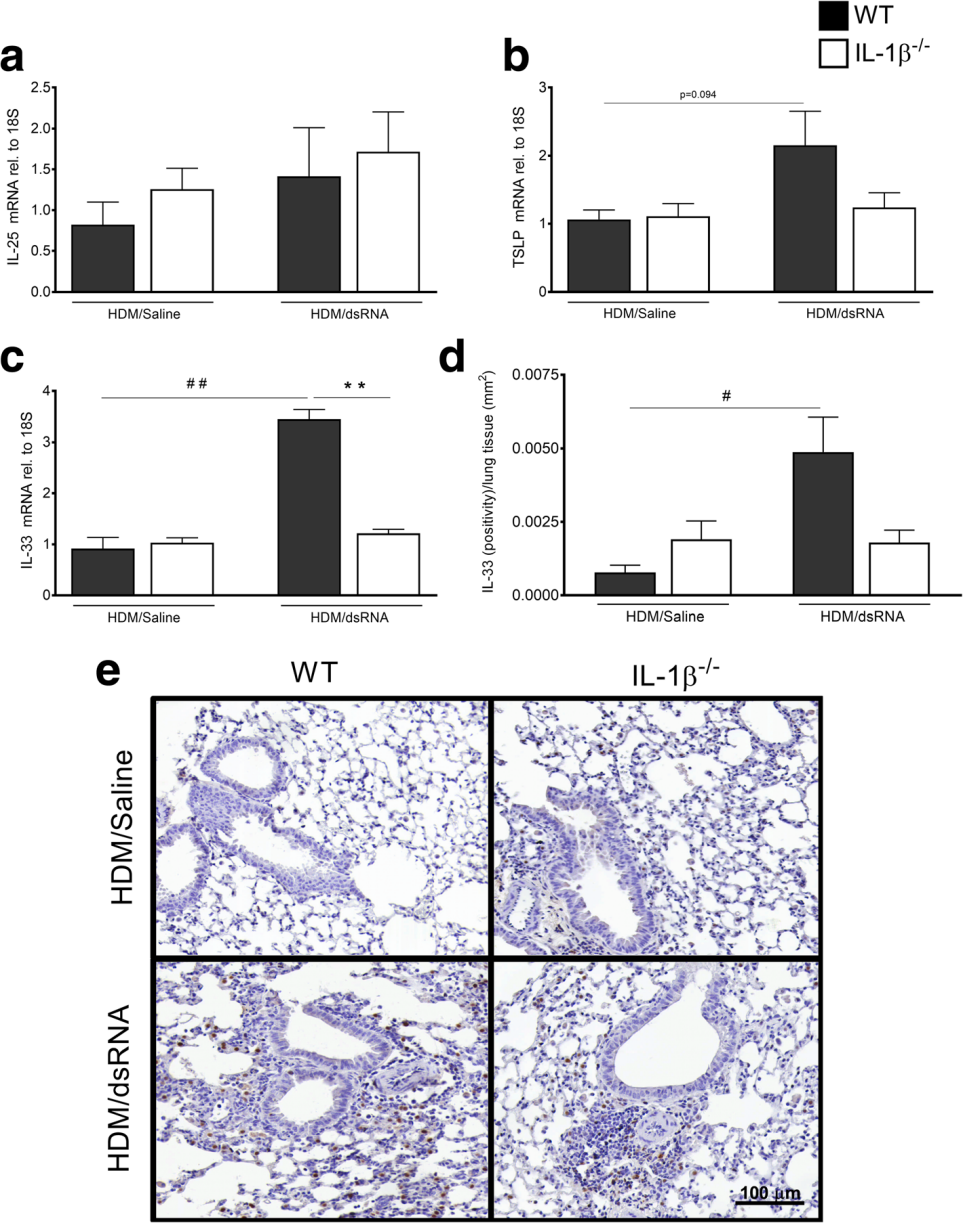
Regulatory role of IL-1β at exacerbation in IL-33 induction in lung tissue*.* mRNA expression of IL-25 (**a**), TSLP (**b**) and IL-33 (**c**) was analysed in lung tissue homogenates by RT-qPCR. The relative gene expression was related to the reference gene 18S and normalised to the fold change of HDM/Saline control. The staining of IL-33 protein by immunohistochemistry was quantified and presented as positivity in tissue area (**d**), also IL-33 stained representative photos shown for each treatment group (**e**). Scale bar indicates 100 μm for all four micrograph images. Data are presented as mean ± SEM, *n* = 4–8 mice in each group. ## = *p* < 0.01 compared to respective HDM/Saline control and # = *p* < 0.05 compared to respective HDM/Saline control. The comparison between WT and IL-1β^−/−^ at exacerbation is indicated by ** = *p* < 0.01

Expression of the Th2-upstream cytokines, IL-25, TSLP and IL-33, was assessed in lung tissue homogenates (Fig. 4a-c). IL-25 gene expression was not elevated at exacerbation and did not seem to be affected by the knockdown of IL-1β (Fig. 4a). The gene expression of TSLP (Fig. 4b) and IL-33 (Fig. 4c) was induced in WT mice at exacerbation with a particularly significant increase seen with IL-33 (*p* < 0.01). By contrast, the expression was not altered in the IL-1β^−/−^ mice (Fig. 4b-c). We continued to analyse IL-33 protein in lung tissue sections visualised by immunohistochemistry staining technique. The relative amount of IL-33 protein present in the stained lung sections was significantly increased at exacerbation in the WT mice but unaltered in the IL-1β deficient group (Fig. 4d-e). There was no tendency at all that lack of IL-1β brought any reduction in Th2-upstream cytokines in animals receiving saline post HDM exposure (Fig. 4).

## Discussion

The present study involved WT and IL-1β-deficient mice subject to HDM-induced experimental asthma with added viral stimulation-induced exacerbation. The experimental exacerbation of asthma exhibited IL-1β-dependent features of neutrophilic lung inflammation as well as Th2-upstream cytokines. Absence of IL-1β did not seem to affect the baseline HDM-induced allergic effects. These data are novel with respect to in vivo experimental asthma exacerbations and forward IL-1β as a potential target in viral infection-induced episodes of asthma that currently are not well treated.

Several aspects were considered for the choice of an in vivo exacerbation mouse model for exploring IL-1β-dependent responses. The basic allergic inflammation model of asthma should preferably involve a human asthma relevant allergen that produces lung eosinophilic inflammation without the need of an adjuvant. HDM extract has these features and is preferred over the commonly employed OVA, which is an allergen irrelevant for human asthma that may not produce inflammation without adjuvant. The rhinoviral stimulation method to produce exacerbation is not as obvious as the choice of allergen. Approaches have been employed using RV1B infection but using a baseline inflammation produced by OVA [20]. Studies employing HDM and RV1B infection have been carried out [19], but as reported by Rochlitzer et al., no exacerbation could be detected [21]. As an alternative to rhinoviral infection, Poly(I:C) has been forwarded as an experimental challenge agent, which is a synthetic form of the dsRNA that is produced at rhinoviral replication and mimics biological effects of rhinovirus infection [22]. dsRNA is sensed by the PRRs Toll-like receptor 3 (TLR3) or the retinoic acid inducible gene-1-(RIG-I)-like receptors [23, 24]. Furthermore, dsRNA-challenges represent a given pathogen burden, which is desirable in studies comparing responses to viral stimulus-induced effects. Thus, dsRNA challenges produce robust responses independent of factors such as take rate that may cause variation in experiments involving actual infection. Synthetic dsRNA has also been successfully employed to produce exacerbation effects in mice with established HDM-induced pulmonary inflammation [25]. Thus, increased eosinophilic and neutrophilic inflammation was induced together with general signs of inflammation including increased BALF levels of LDH [6], a typical feature of viral-induced human asthma exacerbations [5]. In addition, this study demonstrated effects consistent with the functional pathophysiology of exacerbations. Thus, our finding of increased expression of lung Muc5a agrees with mucus hypersecretion at asthma exacerbations [26]. We also confirmed our previous observation of increased total protein likely reflecting plasma exudation, which is a major sign of exacerbations in human asthma [17].

IL-1β, may not have been extensively studied in relation to exacerbations of asthma. However, human bronchial epithelial cells are known to produce IL-1β, in response to RV infection [27]. It has also been demonstrated that experimental RV infection of human nasal airways causes increased IL-1β, immune-reactivity associated with protein exudation and symptoms [28]. We demonstrated increased lung expression of IL-1β, mRNA at exacerbations in WT animals (this study) supporting its potential participation as pathogenic factor. In the present model, HDM alone did not increase IL-1β, above that of KO animals. Indeed, the general picture in this study is that IL-1β, is involved in the viral stimulus-induced exacerbation but not in the allergic responses to HDM.

A neutrophilic nature of viral induced asthma exacerbations is established [5, 13]. Lung neutrophilia is also a conspicuous feature of the present in vivo model. In human asthma sputum neutrophil elastase is increased at exacerbations and considered to be potentially involved in mucin hypersecretion [13], a possibility that might apply to the present model. A major neutrophil chemokine, CXCL1 (both gene expression and protein production), was markedly increased at exacerbation in WT mice compared to the IL-1β deficient mice. Also, this decisive role of IL-1β in CXCL1 secretion was exclusively observed at exacerbation and not by allergen challenge alone. Mouse CXCL1 corresponds to human IL-8 that is increased in exacerbating asthmatic airways [13]. In addition, we recorded increased expression of other chemokines with known neutrophil attractant properties including RANTES/CCL5 [29], in lungs of exacerbating WT animals but not in IL-1β-deficient mice. With the present size of test groups, the reduced chemotactic factors in KO animals were associated with effects on the neutrophilia of exacerbations that only bordered on significance. At exacerbation the KO animals thus tended to have reduced BALF neutrophilia (*p* = 0.06) whereas little anti-neutrophil effects were seen in lung tissue. Thus, further work is warranted to determine the degree of IL-1β, dependent neutrophilic inflammation at viral induced exacerbations including demonstrations of specific pathogenic roles of lung tissue and lumen neutrophils in this condition.

IL-1β-neutralising biologicals such as anakinra and canakinumab are available. However, little is known about their effects in human asthma beyond an early study indicating that anakinra may attenuate allergen challenge-induced late phase reaction [30]. Limited efficacy together with an increased risk for infections with such broadly active inhibitors may be a reason behind discontinued evaluation of these biologicals in different types of asthma. The present data do not support a role of IL-1β in allergic asthma since inflammatory effects of HDM alone were not reduced in IL-1β-deficient mice. By contrast, the present data suggested involvement of IL-1β, at viral induced asthma exacerbations. Currently, there is little information on any clinical effects of IL-1β active drugs on asthma exacerbations.

At exacerbation deficiency in IL-1β was associated with reduced features of neutrophilic inflammation as well as a tendency towards reduced induction of TSLP gene expression whereas IL-33 gene levels were significantly inhibited. These two cytokines have proposed upstream roles in viral induced exacerbation of asthma [4, 31]. The present demonstration of increased lung IL-33 gene expression and protein at viral stimulus-induced exacerbation in WT mice with HDM-induced asthma significantly extends our previous observation on IL-33 expression in this model [6]. A recent study, involving both experiments in patients and studies using cultured bronchial epithelial cells, has singled out IL-33 as a key mechanistic link between viral infection and exacerbations of asthma [4]. Hence, the present demonstration of inhibition of lung IL-33 expression along with a trend at reduced protein at exacerbation in IL-1β deficient mice may be important. There is interest in a role of IL-1β as inducer of IL-17 and IL-6 in asthma. However, since antagonising biologicals have failed to produce acceptable clinical effects, phenotypes or endotypes of asthma where these cytokines have significant roles have yet to be identified [32]. Based on the present data we suggest that IL-1β is a participating factor in viral induced asthma exacerbations but not in baseline allergic conditions. Indeed, our data suggest that by targeting IL-1β, both Th2 and neutrophilic components of asthma exacerbations may be reduced.

The present study is an exploratory in vivo approach and as such harbours a range of limitations. Several of our findings are novel findings. Even if statistically significant they need confirmation in future studies. This concerns unexpected observations of particular interest including potential roles of IL-1β, in Th2 aspects of viral induced exacerbations yet lack of a role in allergic mouse asthma. Human asthma exacerbations exhibit Th2 features. Hence, the present observations on possible involvement of upstream Th2 cytokines in exacerbations are of interest. However, we have not studied whether further Th2 cytokines are actually produced and what roles they may have. Our model of exacerbation has robust features, which have influenced the present design including the size of treatment groups. The intervention data that we now report provide additional information of importance for future approaches. Thus, extended groups seem required to learn whether effects, where we now see a trend, will prove to involve significant information. This latter limitation concerns involvement of IL-1β, in both the neutrophilic and the Th2 features of viral stimulus-induced exacerbations. Future studies are also warranted to validate our findings at exacerbations involving actual rhinoviral infection.

## Conclusion

The present data obtained in WT and IL-1β-deficient mice suggest that viral induced asthma exacerbation in part may depend on IL-1β, whereas baseline HDM-induced experimental asthma may not. Thus, IL-1β signalling emerged as causally involved in expression of Muc5a, a mucus secretion index, CXCL1, a neutrophil chemotactic cytokine, and IL-33, a Th2-upstream cytokine exclusively at exacerbation. We suggest that IL-1β signalling pathways may be causally involved in induction of neutrophilic and Th2 features of viral induced asthma exacerbations.

## Additional files

Additional file 1: Figure S1. IL-1β mRNA expression induced at exacerbation in the WT mice while completely abolished in both IL-1β^−/−^ groups. mRNA was measured from lung tissue homogenates by RT-qPCR. The relative gene expression was related to the reference gene 18S and normalised to control. Data are presented as mean ± SEM, *n* = 4–7 mice in each group. ## = *p* < 0.01 compared to respective HDM/Saline control and comparison between WT and IL-1β^−/−^, at exacerbation is indicated by ** = *p* < 0.01. (TIFF 542 kb)

Additional file 2: Table S1. Commercially available primer sequences for genes analysed with RT-qPCR. (DOCX 13 kb)

Additional file 3: Figure S2. Increased immune cells and total protein in BALF in both WT and IL-1β^−/−^ groups 24 h after the last HDM challenge alone. Total protein in BALF (a), total cells from BALF (b) and total neutrophil count in BALF (c). Data are presented as mean ± SEM, n = 4–7 mice in each group. ## = *p* < 0.01 compared to respective HDM/Saline control, and # = *p* < 0.05 compared to respective HDM/Saline control. (TIFF 941 kb)

Additional file 4: Figure S3. Increased immune cell count and chemokine expression at exacerbation, although in both WT and IL-1β^−/−^ mice. Total eosinophil count (a), macrophage count (b) and lymphocyte count (c) from BALF at exacerbation. Chemokine mRNA expression of CCL11 (d) and CCL2 (e) from lung tissue homogenates were analysed with RT-qPCR. The relative gene expression was related to the reference gene 18S and normalised to control. Data are presented as mean ± SEM, *n* = 4–8 mice in each group. ## = *p* < 0.01 compared to respective HDM/Saline control. (TIFF 1200 kb)

## Abbreviations

TNF-α: Tumor necrosis factor-alpha
18S: 18 Svedberg units (ribosomal RNA)
BALF: Bronchoalveolar lavage fluid
CCL: Chemokine (C-C motif) ligand
COPD: Chronic obstructive pulmonary disease
CXCL: Chemokine (C-X-C motif) ligand
dsRNA: double stranded ribonucleic acid
ELISA: Enzyme-linked immunosorbent assay
GAPDH: Glyceraldehyde 3-phosphate dehydrogenase
HDM: House dust mite
IL: Interleukin
KC: Keratinocyte derived chemokine
KO: Knockout
LDH: Lactate Dehydrogenase
MCP-1: Monocyte chemoattractant protein-1
mRNA: messenger ribonucleic acid
NLRP3: nod-like receptor family, pyrin domain containing-3
OVA: Ovalbumin
Poly(I:C): Polyriboinosinic:polyribocytidylic acid
PRR: Pattern recognition receptor
RANTES: Regulated on activation, normal T cell expressed and secreted
RIG-I: Retinoic acid inducible gene-1
RT-qPCR: Reversed transcription-quantitative polymerase chain reaction
Th2: T helper type 2
TLR3: Toll-like receptor 3
TSLP: Thymic stromal lymphopoietin
WT: wild-type

## References

[1] Busse WW, Lemanske RF, Gern JE (2010). Role of viral respiratory infections in asthma and asthma exacerbations. Lancet.

[2] Jackson DJ, Johnston SL (2010). The role of viruses in acute exacerbations of asthma. J Allergy Clin Immunol.

[3] Persson C, Uller L (2013). HH salter (1860s): taking cold as original cause and provocative of attacks of asthma. Thorax.

[4] Jackson DJ, Makrinioti H, Rana BM, Shamji BW, Trujillo-Torralbo MB, Footitt J, Jerico D-R, Telcian AG, Nikonova A, Zhu J (2014). IL-33-dependent type 2 inflammation during rhinovirus-induced asthma exacerbations in vivo. Am J Respir Crit Care Med.

[5] Wark PA, Johnston SL, Moric I, Simpson JL, Hensley MJ, Gibson PG (2002). Neutrophil degranulation and cell lysis is associated with clinical severity in virus-induced asthma. Eur Respir J.

[6] Mahmutovic Persson I, Akbarshahi H, Menzel M, Brandelius A, Uller L (2016). Increased expression of upstream TH2-cytokines in a mouse model of viral-induced asthma exacerbation. J Transl Med.

[7] Mahmutovic-Persson I, Akbarshahi H, Bartlett NW, Glanville N, Johnston SL, Brandelius A, Uller L (2014). Inhaled dsRNA and rhinovirus evoke neutrophilic exacerbation and lung expression of thymic stromal lymphopoietin in allergic mice with established experimental asthma. Allergy.

[8] Rossios C, Pavlidis S, Hoda U, Kuo CH, Wiegman C, Russell K, Sun K, Loza MJ, Baribaud F, Durham AL, et al. Sputum transcriptomics reveal upregulation of IL-1 receptor family members in patients with severe asthma. J Allergy Clin Immunol. 2017;30764-9. 10.1016/j.jaci.2017.02.045. 10.1016/j.jaci.2017.02.045 28528200

[9] Fu JJ, McDonald VM, Baines KJ, Gibson PG (2015). Airway IL-1beta and Systemic inflammation as predictors of future exacerbation risk in asthma and COPD. Chest.

[10] Pinkerton JW, Kim RY, Robertson AA, Hirota JA, Wood LG, Knight DA, Cooper MA, O’Neill LA, Horvat JC, Hansbro PM. Inflammasomes in the lung. Mol Immunol. 2017;86:44-55. 10.1016/j.molimm.2017.01.014. 10.1016/j.molimm.2017.01.014 28129896

[11] Jo EK, Kim JK, Shin DM, Sasakawa C (2016). Molecular mechanisms regulating NLRP3 inflammasome activation. Cell Mol Immunol.

[12] Kim RY, Pinkerton JW, Essilfie AT, Robertson AAB, Baines KJ, Brown AC, Mayall JR, Ali MK, Starkey MR, Hansbro NG (2017). Role for NLRP3 Inflammasome-mediated, IL-1beta-dependent responses in severe, steroid-resistant asthma. Am J Respir Crit Care Med.

[13] Fahy JV, Kim KW, Liu J, Boushey HA (1995). Prominent neutrophilic inflammation in sputum from subjects with asthma exacerbation. J Allergy Clin Immunol.

[14] Nomura K, Kojima T, Fuchimoto J, Obata K, Keira T, Himi T, Sawada N (2012). Regulation of interleukin-33 and thymic stromal lymphopoietin in human nasal fibroblasts by proinflammatory cytokines. Laryngoscope.

[15] Ambite I, Puthia M, Nagy K, Cafaro C, Nadeem A, Butler DS, Rydstrom G, Filenko NA, Wullt B, Miethke T, Svanborg C (2016). Molecular basis of acute cystitis reveals susceptibility genes and immunotherapeutic targets. PLoS Pathog.

[16] Livak KJ, Schmittgen TD (2001). Analysis of relative gene expression data using real-time quantitative PCR and the 2(−Delta Delta C(T)) method. Methods.

[17] Persson C, Uller L (2009). Roles of plasma exudation in asthma and COPD. Clin Exp Allergy.

[18] Fujisawa T, Velichko S, Thai P, Hung LY, Huang F, Wu R (2009). Regulation of airway MUC5AC expression by IL-1beta and IL-17A; the NF-kappaB paradigm. J Immunol.

[19] Gray T, Nettesheim P, Loftin C, Koo JS, Bonner J, Peddada S, Langenbach R (2004). Interleukin-1beta-induced mucin production in human airway epithelium is mediated by cyclooxygenase-2, prostaglandin E2 receptors, and cyclic AMP-protein kinase a signaling. Mol Pharmacol.

[20] Bartlett NW, Walton RP, Edwards MR, Aniscenko J, Caramori G, Zhu J, Glanville N, Choy KJ, Jourdan P, Burnet J (2008). Mouse models of rhinovirus-induced disease and exacerbation of allergic airway inflammation. Nat Med.

[21] Rochlitzer S, Hoymann HG, Muller M, Braun A, Consortium UB (2014). No exacerbation but impaired anti-viral mechanisms in a rhinovirus-chronic allergic asthma mouse model. Clin Sci (Lond).

[22] Ritter M, Mennerich D, Weith A, Seither P (2005). Characterization of toll-like receptors in primary lung epithelial cells: strong impact of the TLR3 ligand poly(I:C) on the regulation of toll-like receptors, adaptor proteins and inflammatory response. J Inflamm (Lond).

[23] Alexopoulou L, Holt AC, Medzhitov R, Flavell RA (2001). Recognition of double-stranded RNA and activation of NF-kappaB by toll-like receptor 3. Nature.

[24] Takahasi K, Yoneyama M, Nishihori T, Hirai R, Kumeta H, Narita R, Gale M, Inagaki F, Fujita T (2008). Nonself RNA-sensing mechanism of RIG-I helicase and activation of antiviral immune responses. Mol Cell.

[25] Clarke DL, Davis NH, Majithiya JB, Piper SC, Lewis A, Sleeman MA, Corkill DJ, May RD (2014). Development of a mouse model mimicking key aspects of a viral asthma exacerbation. Clin Sci (Lond).

[26] Wark PA, Gibson PG (2006). Asthma exacerbations. 3: pathogenesis. Thorax.

[27] Shi L, Manthei DM, Guadarrama AG, Lenertz LY, Denlinger LC (2012). Rhinovirus-induced IL-1beta release from bronchial epithelial cells is independent of functional P2X7. Am J Respir Cell Mol Biol.

[28] Proud D, Gwaltney JM, Jr., Hendley JO, Dinarello CA, Gillis S, Schleimer RP: Increased levels of interleukin-1 are detected in nasal secretions of volunteers during experimental rhinovirus colds**.** J Infect Dis 1994, 169**:**1007–1013. 10.1093/infdis/169.5.1007 8169385

[29] Pan ZZ, Parkyn L, Ray A, Ray P (2000). Inducible lung-specific expression of RANTES: preferential recruitment of neutrophils. Am J Physiol Lung Cell Mol Physiol.

[30] Rogliani P, Calzetta L, Ora J, Matera MG (2015). Canakinumab for the treatment of chronic obstructive pulmonary disease. Pulm Pharmacol Ther.

[31] Uller L, Leino M, Bedke N, Sammut D, Green B, Lau L, Howarth PH, Holgate ST, Davies DE (2010). Double-stranded RNA induces disproportionate expression of thymic stromal lymphopoietin versus interferon-beta in bronchial epithelial cells from donors with asthma. Thorax.

[32] Peebles RS (2017). Is IL-1beta inhibition the next therapeutic target in asthma?. J Allergy Clin Immunol.

