# Supplementary figures and images for: IL-1β mediates lung neutrophilia and IL-33 expression in a mouse model of viral-induced asthma exacerbation
Source: Respir Res. 2018 Jan 24;19:16. doi: 10.1186/s12931-018-0725-z (PMC5781288; doi:10.1186/s12931-018-0725-z)

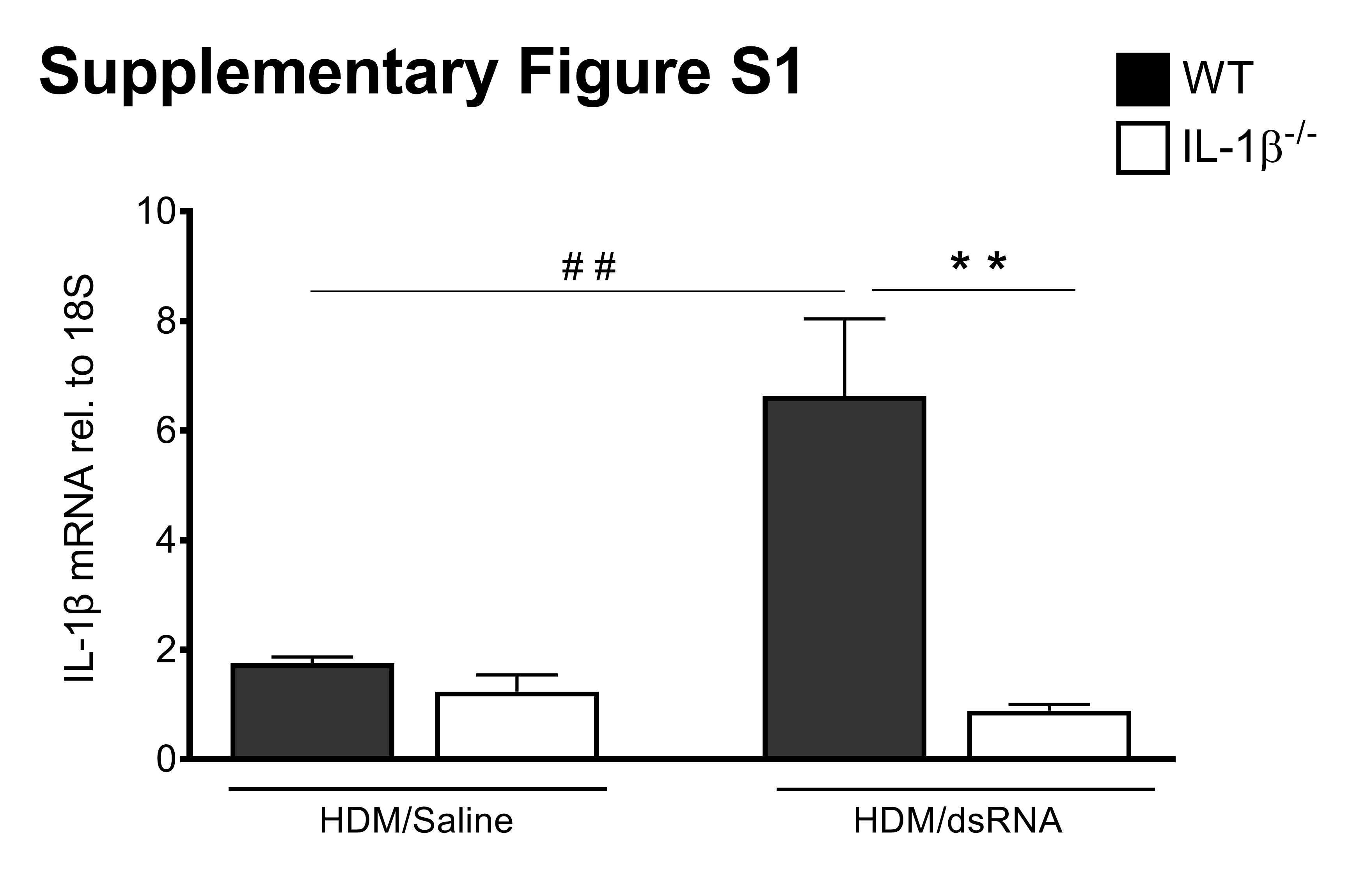

Supplement: Supplementary file 1 — IL-1β mRNA expression induced at exacerbation in the WT mice while completely abolished in both IL-1β−/− groups. mRNA was measured from lung tissue homogenates by RT-qPCR. The relative gene expression was related to the reference gene 18S and normalised to control. Data are presented as mean ± SEM, n = 4–7 mice in each group. ## = p < 0.01 compared to respective HDM/Saline control and comparison between WT and IL-1β−/−, at exacerbation is indicated by ** = p < 0.01. (TIFF 542 kb) [file 12931_2018_725_MOESM1_ESM.tif]

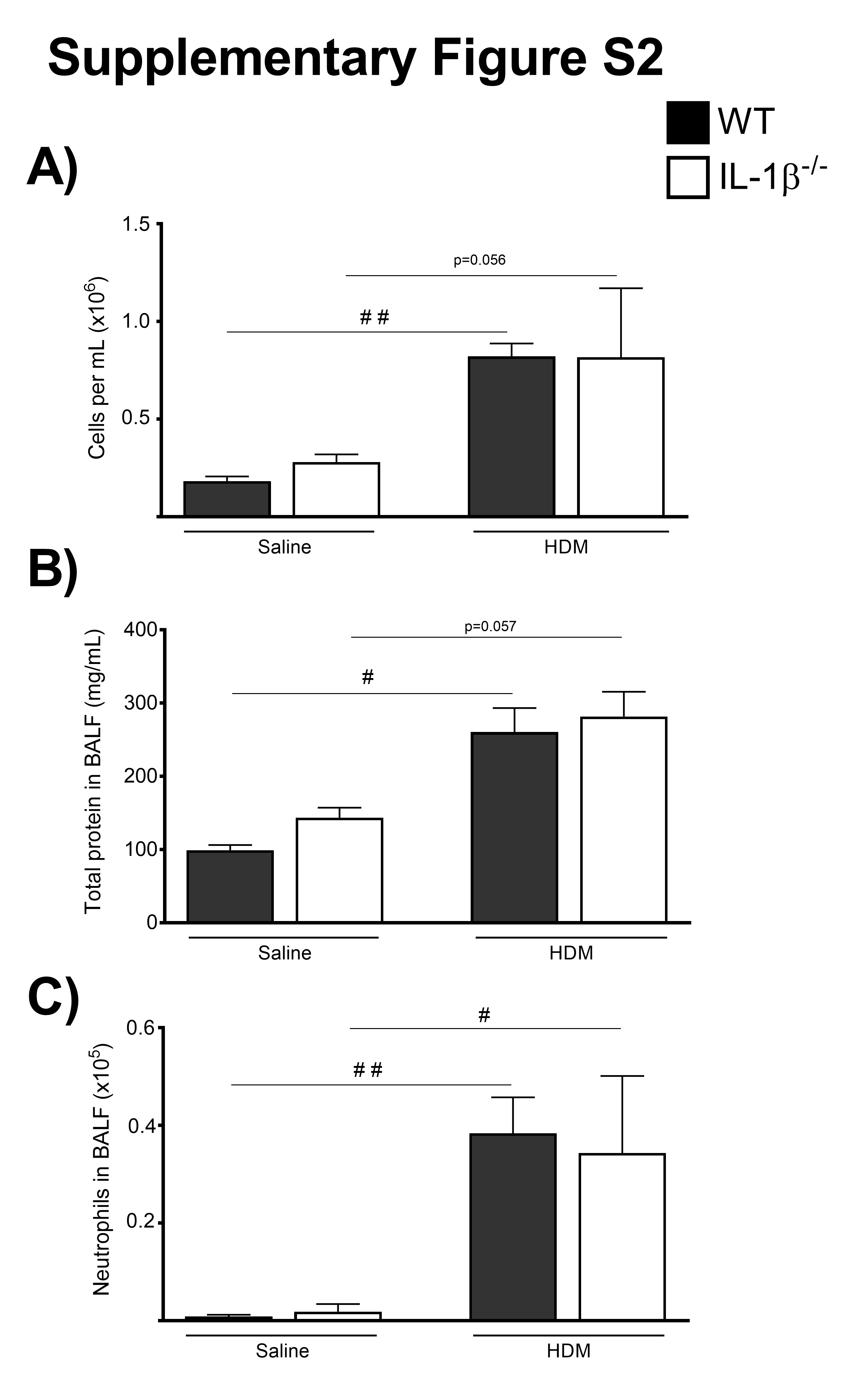

Supplement: Supplementary file 3 — Increased immune cells and total protein in BALF in both WT and IL-1β−/− groups 24 h after the last HDM challenge alone. Total protein in BALF (a), total cells from BALF (b) and total neutrophil count in BALF (c). Data are presented as mean ± SEM, n = 4–7 mice in each group. ## = p < 0.01 compared to respective HDM/Saline control, and # = p < 0.05 compared to respective HDM/Saline control. (TIFF 941 kb) [file 12931_2018_725_MOESM3_ESM.tif]

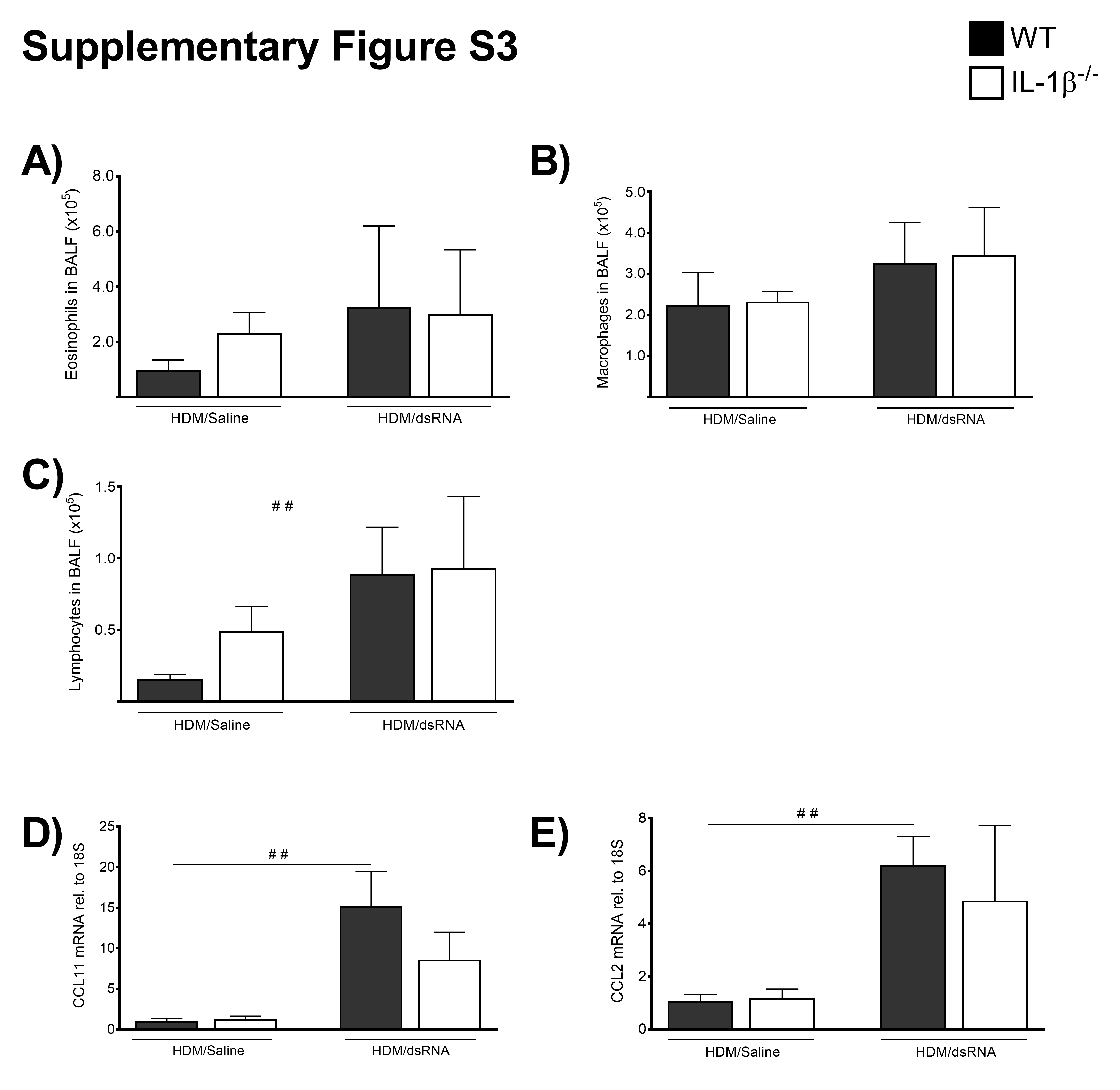

Supplement: Supplementary file 4 — Increased immune cell count and chemokine expression at exacerbation, although in both WT and IL-1β−/− mice. Total eosinophil count (a), macrophage count (b) and lymphocyte count (c) from BALF at exacerbation. Chemokine mRNA expression of CCL11 (d) and CCL2 (e) from lung tissue homogenates were analysed with RT-qPCR. The relative gene expression was related to the reference gene 18S and normalised to control. Data are presented as mean ± SEM, n = 4–8 mice in each group. ## = p < 0.01 compared to respective HDM/Saline control. (TIFF 1200 kb) [file 12931_2018_725_MOESM4_ESM.tif]
