# Supplementary material for: IL-1β mediates lung neutrophilia and IL-33 expression in a mouse model of viral-induced asthma exacerbation
Source: Respir Res. 2018 Jan 24;19:16. doi: 10.1186/s12931-018-0725-z (PMC5781288; doi:10.1186/s12931-018-0725-z)
Supplement: Supplementary file 2 — Commercially available primer sequences for genes analysed with RT-qPCR. (DOCX 13 kb) [file 12931_2018_725_MOESM2_ESM.docx]

**Table S1**. Commercially available primer sequences for genes analysed with RT-qPCR.

**Gene Source Sequence**

Muc5ac QIAGEN Mm_Muc5ac_1_SG (QT01196006) QuantiTect Primer Assay, 102bp

CXCL1 QIAGEN Mm_Ccl5_1_SG (QT00115647) QuantiTect Primer Assay, 93bp

CCL5: QIAGEN Mm_Ccl5_2_SG (QT01747165) QuantiTect Primer Assay, 107bp

IL-25: QIAGEN Mm_Il25_1_SG (QT00134645) QuantiTect Primer Assay, 148bp

IL-1β: QIAGEN Mm_Il1b_2_SG (QT01048355) QuantiTect Primer Assay, 150bp

CCL11: QIAGEN Mm_Ccl11_1_SG (QT00114275) QuantiTect Primer Assay,109bp

---------------------------------------------------------------------------------------------------------------------------

TNF-α: PrimerDesign AGCCAGGAGGGAGAACAGA (forward)

CAGTGAGTGAAAGGGACAGAAC (reverse)

TSLP: PrimerDesign AAACTGAGAGAAATGACGGTACT (forward)

TCTGGAGATTGCATGAAGGAATA (reverse)

IL-33: PrimerDesign CAATGTTGACGACTCTGGAAAAG (forward)

GGGACTCATGTTCACCATCAG (reverse)

CCL2: PrimerDesign TGAAGTTGACCCGTAAATCTGAA (forward)

AGGCATCACAGTCCGAGTC (reverse)
